# Supplementation of *Lactobacillus* early in life alters attention bias to threat in piglets

**DOI:** 10.1038/s41598-021-89560-2

**Published:** 2021-05-12

**Authors:** Else Verbeek, Johan Dicksved, Linda Keeling

**Affiliations:** 1 Department of Animal Environment and Health, Swedish University of Agricultural Sciences, Box 7068, 750 07 Uppsala, Sweden; 2 Department of Animal Nutrition and Management, Swedish University of Agricultural Sciences, Box 7024, 750 07 Uppsala, Sweden

**Keywords:** Animal behaviour, Agroecology

## Abstract

Gut microbes play an important role in regulating brain processes and influence behaviour, cognition and emotional states in humans and rodents. Nevertheless, it is not known how ingestion of beneficial microbes modulates emotional states in piglets and whether it can improve welfare. Here we use an attention bias task to assess the effects of *Lactobacillus reuteri* ATCC-PTA-6475 and *Lactobacillus plantarum* L1-6 supplementation early in life on emotional states in 33 piglets compared to 31 placebo supplemented piglets. We hypothesized that *Lactobacillus* supplementation would reduce vigilance behaviour (head at shoulder height or higher) and attention (head oriented towards the threat) in response to an auditory threat. The results showed that the control group increased vigilance behaviour in response to the threat, but there was no increase in the probiotics group. Despite the increased vigilance, the control group paid less attention to the threat. One explanation may be that control piglets avoided looking in the direction of the threat just because they perceived it as more threatening, but further research is necessary to confirm this. In conclusion, *Lactobacillus* supplementation may be a suitable tool to reduce anxiety, promote a more appropriate response to a challenge and so improve welfare.

## Introduction

The intestine contains trillions of microbes that form an ecosystem commonly referred to as the gut microbiota^1^. A key feature of the gut microbiota is its bidirectional communication with the brain, also called the microbiota-gut-brain axis, through which it can influence fundamental brain processes^2^. The microbial colonization of the gut starts at birth, and the gut microbiota goes through major developmental changes during the first years of life (the first 5–12 years in humans^3,4^ and the first 2–6 months in pigs^5,6^), while the adult microbiota is relatively stable^7^. It is critical to establish a balanced and diverse gut microbiota early in life to guarantee the normal development of several homeostatic processes, including the immune system, the cardiovascular system, the digestive system and metabolic processes^1^. However, the early establishment of the gut microbiota is vulnerable to disturbances, such as a suboptimal diet and environment, use of antibiotics and excessive stress^8^.

The intestinal microbes present early in life play a role in normal brain development^9,10^. The main pathways of microbiota-gut-brain communication are through the central and enteric nervous systems^11,12^, the immune system^13^, the neuro-endocrine system^14^ and through the production of microbial metabolites^15–17^. Mice raised without a gut microbiota (germ-free) showed exaggerated corticosterone and ACTH responses to restraint stress compared to normal mice^10^. Germ-free mice also showed reduced anxiety-like behaviours and increased motor activity^9,18^. However, the reduced anxiety in germ-free mice could be normalized by restoring the gut microbiota post-weaning^19^. In humans, there is now substantial evidence that an unbalanced gut microbiota contributes to the development of a range of abnormal behaviours and can be a contributing factor to depression and anxiety disorders^8,20,21^.

The normal development of the gut microbiota may be compromised in animals raised in indoor environments under strict hygienic conditions, due to a lack of exposure to environmental microbes^22,23^. Furthermore, intensively reared animals, such as pigs, experience multiple stressors already from an early age (e.g., weaning and separation from the dam, castration, frequent mixing with unfamiliar animals). The combination of strict hygienic conditions, multiple early life stressors and use of antibiotics may pose an increased risk of developing an unbalanced gut microbiota in intensively reared animals^8^.

One way to promote a healthy gut development is to supplement the diet with probiotics, defined as live microorganisms that confer a health benefit on the host^24^. For example, supplementing with *Lactobacillus* strains or other beneficial microbes at weaning in piglets has been shown to prevent health problems associated with weaning^25^. In other animal species, several *Lactobacillus* strains are known to influence the behaviour of their host^26^. *Lactobacillus plantarum* supplementation has been shown to reduce anxiety- and depression-like behaviours in rodents^27–29^. Supplementation with *Lactobacillus reuteri* reduced stress-induced anxiety-like behaviours^30^ and restored disturbed social behaviour in mice with an unbalanced gut microbiota^31^. Meta-analyses also suggest that probiotic supplementation in humans can reduce subjective stress levels without altering cortisol levels^32^ and has antidepressant and anxiolytic effects^33^. One possible pathway by which *Lactobacillus* may influence the behaviour of its host is through the production of neurochemicals similar to those produced in vertebrate organisms^34^. *L. reuteri* and *L. plantarum* have been shown to modulate GABAergic and serotonergic signalling pathways in multiple brain regions^28,29,35,36^*.*

It is now widely accepted that the emotional state is a main component of animal welfare^37^. Rodent models and human studies have demonstrated the importance of supporting a normal development of the gut microbiota to promote both physical and mental health^20^, but research into the microbiota-gut-brain axis in other animal species is limited^38^. The pig gut microbiome shares more similarities with the human microbiome than does the mouse microbiome^39^, and therefore the pig is a suitable model to explore the gut-brain axis. In addition, it has already been shown that the emotional state of intensively reared animals is more negative compared to animals living in enriched environments^40,41^. Therefore, elucidating how gut microbes modulate emotional states in pigs may provide an easily applicable tool to improve the welfare of intensively reared animals, and this may also be relevant for human studies. Because both *L. reuteri* and *L. plantarum* have been shown to have a beneficial impact on behaviour^27–29,31^ and alter GABAergic and serotonergic signalling in rodent models^28,29,35,36^, they may be suitable to improve welfare in farmed animals.

Even though animals are not able to communicate their emotions verbally, there are cognitive approaches that can provide insight into animal emotions^42^. One relatively novel approach is to assess changes in attentional processes^43^. Attentional processes are required for the selection of relevant stimuli for further processing because the cognitive system cannot process all sensory stimuli at once^44^. The ability to direct attention efficiently towards situations that enhance or threaten survival provides an adaptive advantage, and this allocation of attentional resources is facilitated by the experience of different emotional states.

Threat signals pose a risk to the animal’s survival and are therefore attended to immediately and automatically, so taking priority over other signals^45^. This effect is further enhanced by negative emotional states: anxious people are quicker to detect a threat and will look at a threat for longer than non-anxious people, which is called an attention bias to threat^46^. Similar attention bias approaches have been developed for animals, in which general vigilance behaviour (i.e., head at shoulder height or higher) and attention directed towards the threat (i.e., the animal looking at the threat) are taken as the main measures of attention^47,48^. Sheep with pharmacologically-induced anxiety showed increased attention towards a dog (predator threat) and increased vigilance behaviour^48^. Stressed starlings were more vigilant after hearing a conspecific alarm call^49^. Pigs with a proactive coping style were more vigilant during a 10 s sudden motion and loud sound stimulus, but no differences in vigilance or attention were found once the threat ended^50^. However, human and primate studies have also shown that the relationship between threat and attention is not linear, and that an initial increased attention towards a threat may be followed by attentional avoidance^51,52^. Together, these studies suggest that attention bias to threat is a promising novel indicator of anxiety in animals^43,52^. Attention bias tasks also have some advantages over more commonly used methods to assess emotions in animals, such as judgement bias, because they require no training and have lower attrition rates^43^, which makes them more suitable for very young (pre-weaning) animals.

Given the critical role of gut microbes in modulating the emotional state and their potential to improve animal welfare, we aim to assess the effects of *Lactobacillus* supplementation early in life on attention bias as an indicator of the emotional state in pre-weaning piglets compared to a placebo control group. We did this by comparing the behavioural responses of individual, supplemented and non-supplemented piglets from the same litter, to a threatening auditory stimulus in a novel test arena. We hypothesize that the *Lactobacillus* supplementation would reduce an anxious emotional state, with supplemented pigs exhibiting reduced attention to a threat and reduced vigilance behaviour following a threat compared to the control group.

## Results

### Vigilance behaviour and attention towards the threat

**Figure 1:**
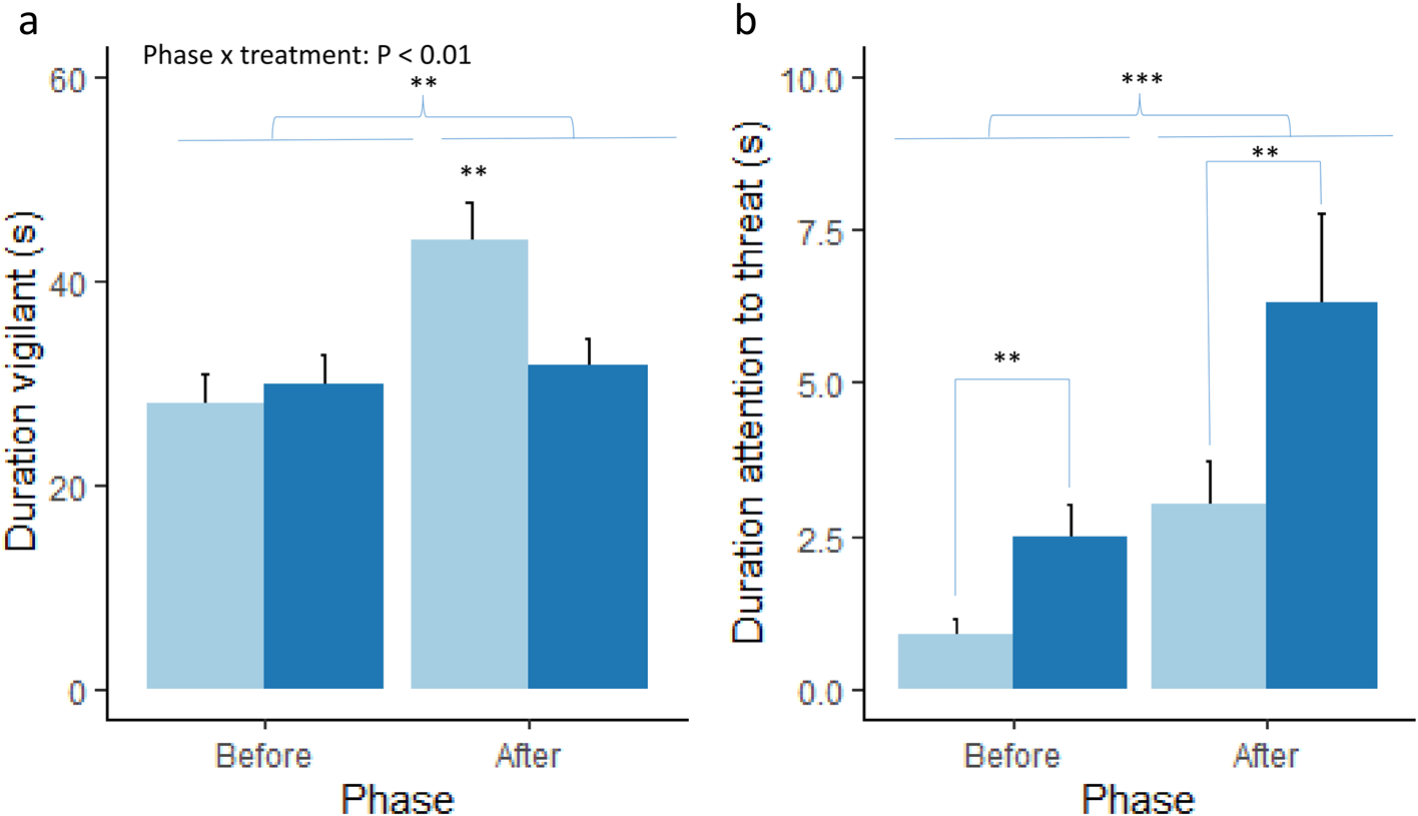
Mean ± standard error of the mean (sem) duration of (**a**) vigilance behaviour and (**b**) attention towards the threat for the control (light blue bars) and probiotic supplemented (dark blue bars) piglets before and after the threat. **p < 0.01, ***p < 0.001.

The duration spent vigilant was significantly affected by a phase x treatment interaction (F_(1,109.02)_ = 8.67, *p* < 0.01, Fig. 1a), with the control piglets increasing the time spent vigilant after the threat while the probiotics supplemented piglets did not (post-hoc Tukey test, *p* < 0.01). Vigilance behaviour was also affected by a test order effect (F_(9,109.28)_ = 3.6, *p* < 0.001), with the animals tested as number 6 in the litter being significantly less vigilant compared to animals tested first (*p* < 0.001) eight, (*p* < 0.05) and ninth (*p* < 0.05). Attention to the threat (Fig. 1b) was significantly affected by phase (Kruskal–Wallis χ^2^ = 12.26, *p* < 0.001) with attention towards the threat increasing after the threat. Attention to threat was also affected by treatment (χ^2^ = 7.31, *p* < 0.01), with the probiotic supplemented piglets paying more attention to the threat.

### Activity and location

**Figure 2:**
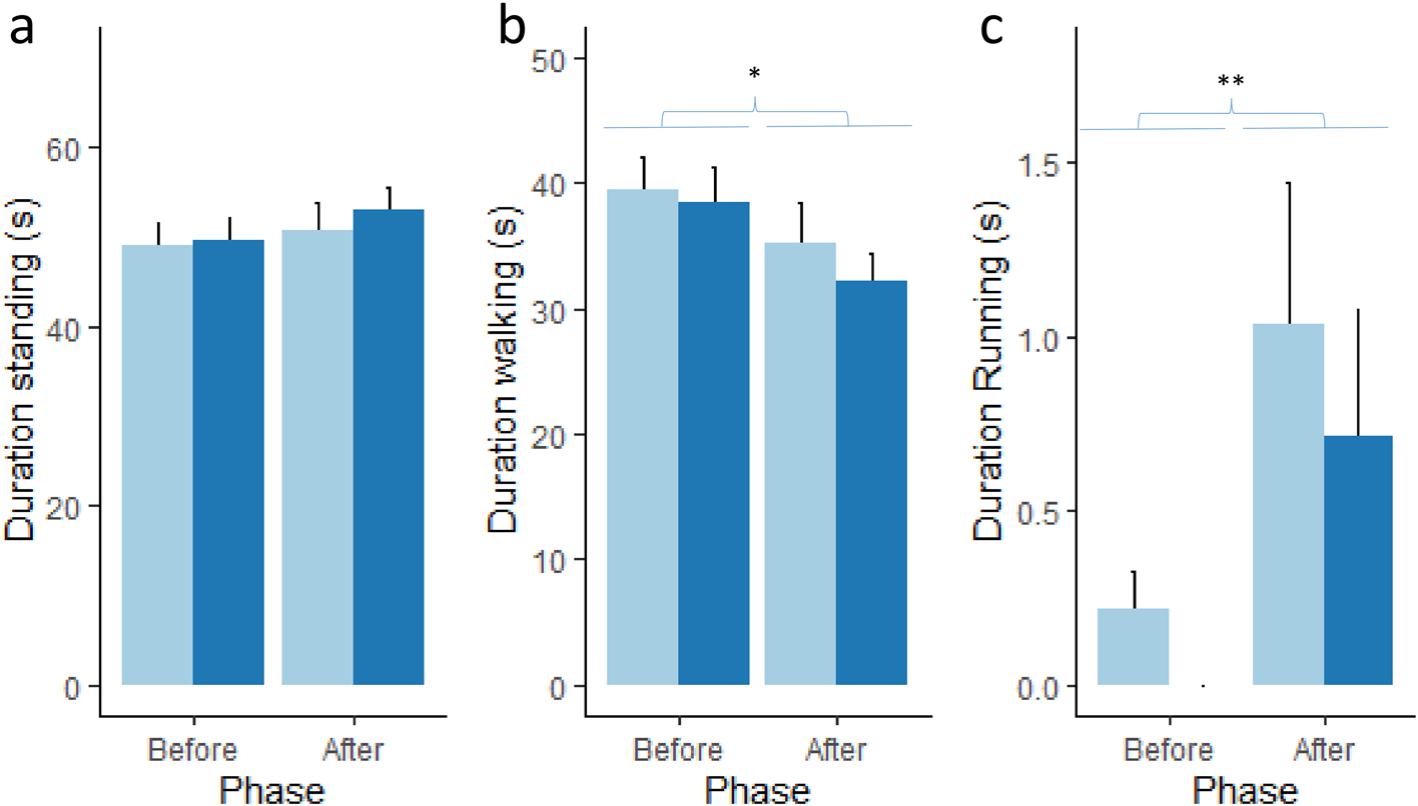
Mean ± sem duration of (**a**) standing behaviour, (**b**) walking behaviour and **c.** running behaviour for the control (light blue bars) and probiotic supplemented (dark blue bars) piglets before and after the threat. *p < 0.05, **p < 0.01.

Piglet activity is shown in Fig. 2. Piglets walked (F_1,115.97_ = 5.6, *p* < 0.05, Fig. 2b) and ran (χ^2^ = 7.06 *p* < 0.01, Fig. 2c) significantly more often after the threat than before the threat. In addition, female piglets (0.93 ± 0.34 s) ran more than male piglets (0.20 ± 0.72 s, χ^2^ = 4.43, *p* < 0.05).

**Figure 3:**
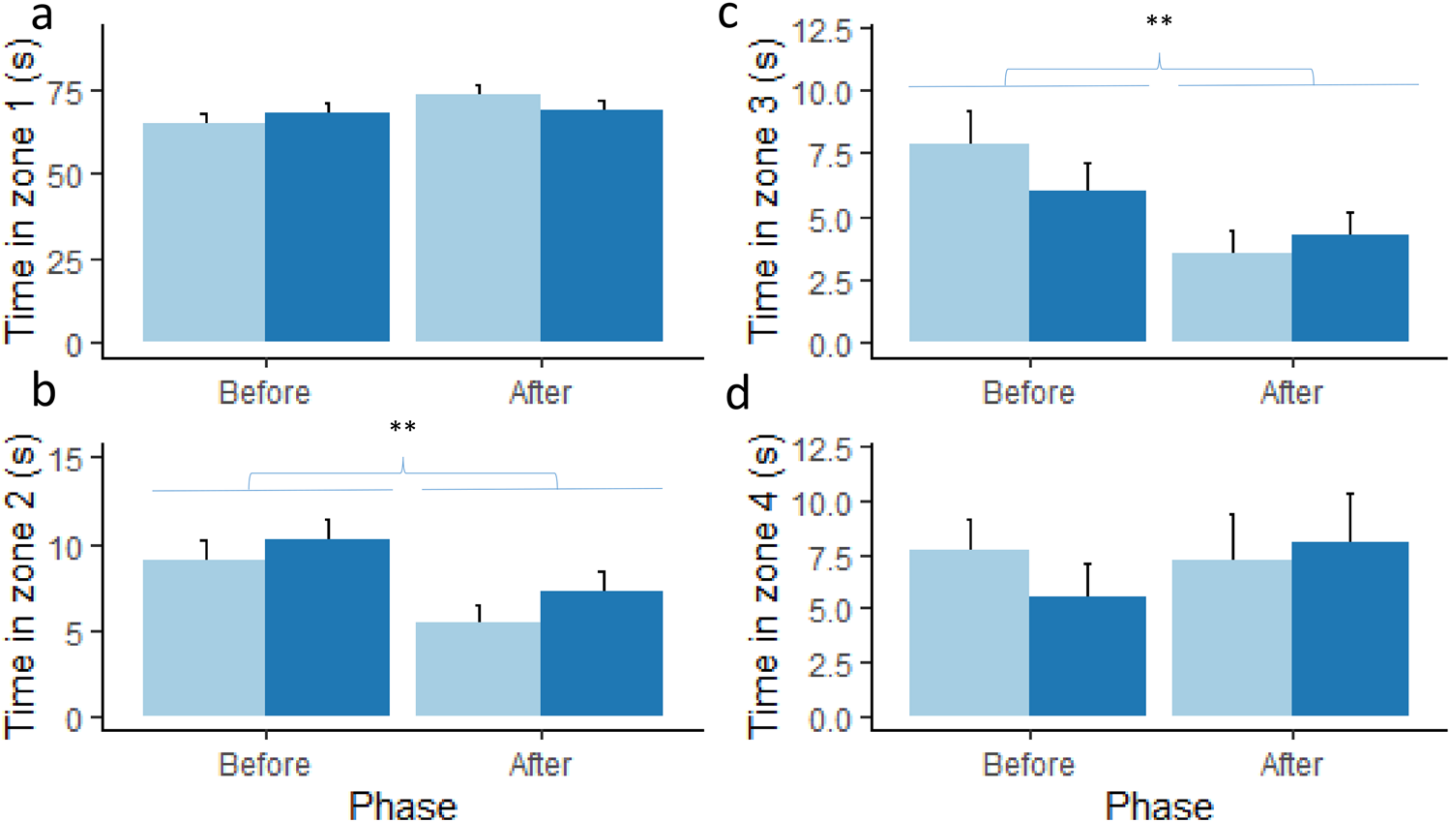
Mean ± sem duration of time spent in (**a**) Zone 1 (area near conspecifics), (**b**) Zone 2 (middle area), (**c**) Zone 3 (middle area) and (**d**) zone 4 (area with substrate and toys) for the control (light blue bars) and probiotic supplemented (dark blue bars) piglets before and after the threat. **p < 0.01.

The time spent in the different zones was not affected by treatment (Fig. 3). Piglets reduced the time spent in zone 2 (χ^2^ = 10.04, *p* < 0.01, Fig. 3b) and 3 (χ^2^ = 8.24, *p* < 0.01, Fig. 3c) after the threat. In addition, male piglets (71.6 ± 2.0 s) spent more time in zone 1 than female piglets (64.8 ± 2.3 s, χ^2^ = 5.51, *p* < 0.05), there were no differences between the sexes for the other zones.

### General behavioural indicators

Other behaviours were also assessed and the statistical parameters are presented in Table 1. There were neither phase nor treatment differences for interacting with the toys, time spent rooting, conspecific directed behaviours or the latency to first contact with the toys. Exploring significantly reduced after the threat (*p* < 0.001) and there was a near-significant tendency for a phase by treatment interaction (*p* = 0.05), with a larger reduction in exploring behaviours in the control piglets after the threat. Females tended to explore (27.9 ± 2 s) more than males (21.1 ± 1.6, F_1,56.6_ = 3.7, *p* = 0.06).

**Table 1:** Mean ± sem behavioural variables during the two different phases of the attention bias test.

Variable	Before		After		Treatment		Phase		Treatment × phase	
	Probiotics	Control	Probiotics	Control	Test value	*p* value	Test value	*p* value	Test value	*p* value
Latency to first reach toys (s)	–	–	100.5 ± 12.8	92.0 ± 13.0	F = 0.108	0.74	–	–	–	–
Time interacting with toys (s)	3.3 ± 0.8	2.4 ± 0.9	4.3 ± 1.1	4.6 ± 1.4	χ^2^ = 0.96	0.33	χ^2^ = 0.40	0.52	–	–
Time spent rooting (s)	0.61 ± 0.5	0.45 ± 0.2	0.29 ± 0.22	0.55 ± 0.27	χ^2^ = 0.62	0.43	χ^2^ = 0.04	0.84	–	–
Time spent exploring (s)	28.4 ± 2.3	28.4 ± 2.3	23.5 ± 3.0	15.9 ± 2.1	F = 1.65	0.2	F = 25.66	< 0.001	F = 3.9	0.05
Conspecific directed behaviours (s)	24.3 ± 2.5	26.9 ± 2.5	25.9 ± 2.7	32.9 ± 3.4	F = 1.9	0.17	F = 2.0	0.15	F = 0.67	0.41

## Discussion

Our results provide the first evidence that *Lactobacillus* supplementation alters anxiety-like states in piglets. An increase in vigilance behaviour in response to an auditory threat was prevented by *Lactobacillus,* and this anxiolytic effect is in agreement with other studies in rodents and humans^20,26^. *Lactobacillus* administered early in life may therefore be a suitable tool to improve the welfare of intensively farmed pigs. It is already common to supplement with *Lactobacillus* or other beneficial microbes around weaning to prevent health problems and increase production in pigs^25^, but our results suggest that there may be a benefit beyond production and health. However, we only assessed attention bias once at 4 weeks of age (pre-weaning) and further studies are necessary to determine any long-term effects.

According to our hypothesis, *Lactobacillus* supplementation prevented an increase in vigilance behaviour following a threat. The *Lactobacillus* and control groups displayed similar levels of vigilance behaviour before the threat, suggesting that the increase in vigilance behaviour in the control group was a direct response to the threat, rather than a more general response. We also detected a test order effect on vigilance behaviour, with animals tested as number six being significantly less vigilant than animals tested first or as number eight or nine in the litter. The reason for this is not clear, and this test order effect was not detected for any other variable. We may speculate that it may be due to the stronger reaction of the conspecifics to the first threat sound, which may have influenced the first test piglet. However, this does not explain the higher vigilance behaviour in pigs tested at number eight and nine in the litter when the conspecifics should have been habituated to the sound. Nevertheless, we had distributed the supplemented and control piglets evenly throughout the testing day, and the treatment of the piglet tested first in the litter was as balanced as possible across the litters. Therefore, despite the test order effect, we could still detect a significant phase by treatment interaction on vigilance behaviour.

Although attention towards the threat was relatively low (less than 10 s compared to around 40 s^48^–70 s^47^ in previous studies in sheep), it increased after the threat in both groups as predicted. However, the lower attention towards the threat in the control group was not according to our prediction and seems counter-intuitive given the increased vigilance behaviour. Paying attention to a threat is adaptive and allows the animal to respond to the threat appropriately^45^. However, attention away from threat stimuli in anxious individuals has also been reported in other studies^53^. Monkeys that had undergone a stressful procedure were more likely to redirect their gaze away from an aggressive monkey face, suggesting a disengagement of attention to threat in stressed animals^52^. Anxious humans also showed attentional avoidance of threatening pictures^54,55^. Therefore, our results may fit the vigilance-avoidance model of attention that has been described in humans^53,56^. This vigilance-avoidance model postulates that anxious people show an increased initial orienting towards a threat, followed by attentional avoidance of it. The attentional avoidance of threatening information may be a way to self-regulate the emotional state^57^ and may play a role in maintaining fear, because it does not allow for habituation to threat stimuli^55,58^. Therefore, a possible explanation for our results could be that the control pigs avoided looking at the threat because they perceived it as more threatening than the supplemented pigs, and consequently did not see there was no actual threat present. They then increased their general vigilance behaviour to be able to respond to a potential threat.

This is the first study, to our knowledge, to investigate the impact of gut-brain axis modification on emotional states in pigs. The pig gut microbiome shares more similarities with the human microbiome than does the mouse microbiome^39^, and therefore the pig is a suitable model to investigate the gut-brain axis. Our results showed that *Lactobacillus* supplementation reduced attention bias towards threat, and future research exploring the therapeutic value of *Lactobacillus* supplementation on reducing anxiety in humans may therefore be warranted.

The *Lactobacillus* supplemented piglets paid more attention to the threat, and this difference already existed before the threat. It is likely that the piglets were paying attention to the observer standing quietly next to the arena (and next to the computer that played-back the threat sound) in the period before the threat. In future studies it would be better if the threat sound came from a different location than the observer, so that attention to the threat and to the observer can be determined independently. Alternatively, the *Lactobacillus* group may have been more attentive to their surroundings in general, but this would need further investigation.

Accurate measures of attention are difficult to record in freely moving animals. We used the direction of the head as a measure of what the animals were looking at—as an indirect indication of attention to threat—but it is likely that we have missed more subtle changes in attention. In humans, eye tracking is commonly used to demonstrate precisely how fast, where and for how long attention is focused^59^, but such technologies have not yet been developed for pigs and would also require some level of restraint. However, what a person is looking at is in most cases also the main focus of attention^44^, and therefore looking towards the threat should have been a reasonable proxy for attention towards the threat. Pigs do not only rely on vision to navigate their environment^60^, and auditory and olfactory cues^61^ are also important. Other more subtle measures of attention, such as automated measurement of the orientation of the ears, could potentially also be used in addition to the head orientation in future studies.

The threat used in this study was a recording of an aggressive dog bark that was most likely unfamiliar to the piglets, because piglets were raised indoors and had never seen a dog. Although we cannot rule out that they may have heard dogs barking outside. Wolves are a natural predator of wild boar, and mostly predate on their piglets^62^, so we reasoned that an aggressive dog bark may trigger a fear response in piglets separated from their mother. However, the novelty of the sound may also have contributed to the observed fear responses. We included a pre-threat and a post-threat period in the attention bias task, in order to separate more general ‘baseline’ behaviours from behavioural responses to a threat, which was an improvement from previously used tasks^47,50,63^. We observed several changes in behaviour between the two different phases. Piglets ran more after the threat, and because increased activity can be interpreted as a sign of fear^64^, this suggests that our threat stimulus indeed induced fear. Females ran more than males across both phases, suggesting that females were either more fearful in general or more active.

Piglets spent less time in the middle of the arena after the threat, potentially because being further away from the conspecifics elicited more fear^65^. However, we did not observe any effect of the *Lactobacillus* on the time spent in the area closest to the conspecifics nor on conspecific directed behaviours between the phases. Males spent more time close to the conspecific than females. The reason for this is not clear, but it could be that males were more fearful, which is in contradiction with the lower activity in males (see above). Alternatively, they could have had had a stronger social motivation than females, although we did not observe any sex differences in conspecific directed behaviours. The piglets spent very little time rooting in the straw area or interacting with the toys and we did not see a difference between the phases. Both the arena and the toys were unfamiliar to the piglets and the toys were on the opposite side from the conspecifics, and piglets may have been too fearful to interact with the substrate and toys. General exploration behaviours (exploration not directed to the substrate or toys) decreased after the threat, especially in the control group. Exploration is a normal and natural behaviour for pigs, and free-range pigs spent a large proportion of their time exploring, rooting and foraging^66^. The larger decrease in exploration behaviour in the control group can probably be explained by their increased vigilance behaviour, and may be indicative of increased fear or anxiety. Females also tended to explore more than males. Previous studies have also shown that 4-week old female piglets spent more time exploring a novel object than males, which the authors suggested was due to faster brain development in females at this age^67^. Other individual factors such as personality traits and state anxiety can also influence fear responses^68^, and it would be interesting to investigate such individual traits on attention bias to threat in future studies.

Within each litter, five piglets were assigned to receive the *Lactobacillus* supplementation and five other piglets received a control treatment. In this way, we could control at least some of the effects attributable to the sow and pen environments, and made it easier for the experimenters to be blind to the treatments. However, this is also means that there could have been some cross-contamination of the *Lactobacillus* strains from the supplemented to the control piglets, that may have diminished some of the differences between the treatments.

In conclusion, this study provides the first evidence that *Lactobacillus* supplementation early in life prevents an increase in vigilance behaviour following a threat in pre-weaning piglets. The control group increased vigilance behaviours in response to the threat but nevertheless reduced orienting in the direction of the threat. One explanation may be that the control piglets avoided looking in the direction of the threat just because they perceived it as more threatening, but further research would be necessary to confirm this. This study provides a good base for further development of *Lactobacillus* supplementation as a tool to promote a more appropriate response to a challenge and improve animal welfare in intensively reared animals.

## Methods

All methods were carried out in accordance with relevant guidelines and regulations. The study was approved by the Uppsala animal ethics committee (document numbers C105416/16 and 5.8.18/01998/2018) and complied with the ARRIVE guidelines^69^.

### Animals and housing

In total, 64 piglets participated in the experiment. The study was conducted at the experimental facilities of the Swedish University of Agricultural Sciences (Lövsta) that houses specific pathogen free sows. Sows were moved to the farrowing unit 1 week before expected farrowing and stayed with their piglets until weaning at 35 days of age. The farrowing pens’ design and size complied with the European animal welfare legislation (Council Directive 2008/120/EC)^70^. The farrowing pens (3.35 × 2.0 m) consisted of a concrete floor lying and feeding area (2.1 m × 2. 0 m), a slatted dung area (1.25 m × 2.0 m), as well as a heated corner that was only accessible to the piglets. Sows were given 15–20 kg of chopped straw two days prior to the expected farrowing date. An additional small amount of straw (0.5–1 kg/day) was given daily as enrichment after the pens were manually cleaned. Sows were fed a standard commercial dry feed for lactating sows by an automatic feeding system (Table 1). The first 10 days, sows were fed twice per day and after that three times a day until weaning. An *ad libitum* creep feed dispenser was accessible to the piglets from two weeks of age and contained a standard piglet creep feed (Table 2). Water was available* ad libitum* from two drinking nipples. Piglets were weighed (birth weight for control piglet 1.71 ± 0.07 kg and supplemented 1.67 ± 0.08 kg) and ear-tattooed with an individual number within 1 day after birth, and received an ear-tag with their individual number at 5 days of age. A 1 mL intramuscular injection of an iron supplement (Uniferon, 200 mg/mL) was given at 5 days and two weeks of age.

**Table 2:** Dietary content for sows and piglets.

Dietary content	Amount	
	Sow	Piglets
Energy (MJ/kg)	13	14.4
NE, MJ	9.9	10.8
Water %	12.3	11.1
Protein (g/kg)	166	285
Fat (g/kg)	58	105
Crude fiber (g/kg)	49	25
Ash (g/kg)	50	85
Sodium (g/kg)	2	3
Potassium (g/kg)	9	17
Lysine (g/kg)	8	17
Metione (g/kg)	3	6
Vitamin A (IE/kg)	8000	16,000
Vitamin D3 (IE/kg)	1600	3320
Vitamin E (IE/kg)	150	400
Selenium (mg/kg)	0.4	0.8

### Experimental design and treatments

Seven sows without any clinical symptoms of disease and their litters were selected directly after birth to participate in the experiment. From each litter, 10 piglets without any clinical symptoms of disease were selected for the experiment. Of these, five piglets were allocated to receive an oral supplement of *Lactobacillus reuteri* ATCC-PTA-6475 (8 × 10^7^ ± 3 × 10^7^ cfu/dose) and *Lactobacillus plantarum* L1-6 (2 × 10^9^ ± 5 × 10^7^ cfu/dose) three times a week, from 3 days of age until weaning at 35 days of age. The remaining five piglets received a control supplement (same media as the supplemented group, but without the *Lactobacillus*) which was administered in the same way as the probiotics supplement. The allocation of the piglets to the two different groups was balanced for weight and sex and much as possible. One researcher (JD) had the responsibility for the preparation of the probiotic and control supplements. The staff at the farm received the supplements labelled with different markings and they provided the supplements to the pigs. None of the farm staff nor the researcher JD were involved in the performance of the attention bias test. At 26.6 ± 0.7 days of age, 31 control piglets (11 females and 20 males) and 33 supplemented piglets (14 females and 19 males) were exposed to one attention bias test in which their behavioural reactions towards and unfamiliar dog bark sound were assessed.

### Preparation of the bacterial strains

*Lactobacillus reuteri* ATCC-PTA-6475 and *Lactobacillus plantarum* L1-6, (kindly provided by Stefan Roos, Swedish University of Agricultural Sciences) were cultured separately in MRS broth (Oxoid Ltd, Basingstoke, England) over night. The microbial cells from the fresh cultures were pelleted by centrifugation at 5000 g for 10 min at 4℃. The supernatants were discarded, the cell pellets re-suspended in saline solution and were again pelleted by centrifugation at 5000 g for 10 min. After discarding the supernatants, the cell pellets were dissolved in a water solution containing 10% sucrose and 1% ascorbic acid. The solutions were homogenized, dispensed in 100 µl aliquots and were stored at -80℃ until use. The levels of bacteria in the prepared bacterial solutions were assessed by plate counting, using MRS agar (Oxoid Ltd).

### Supplementation to pigs

At each supplementation, an aliquot of each bacterial strain (each 100 µl) was thawed and mixed with 20 µl caramel colour and 80 µl water. The solution was supplemented to the piglet via a 1 ml syringe. The control pigs were supplemented with the same amount of caramel colour, water and sucrose solution, but without any bacteria.

### Attention bias test

**Figure 4:**
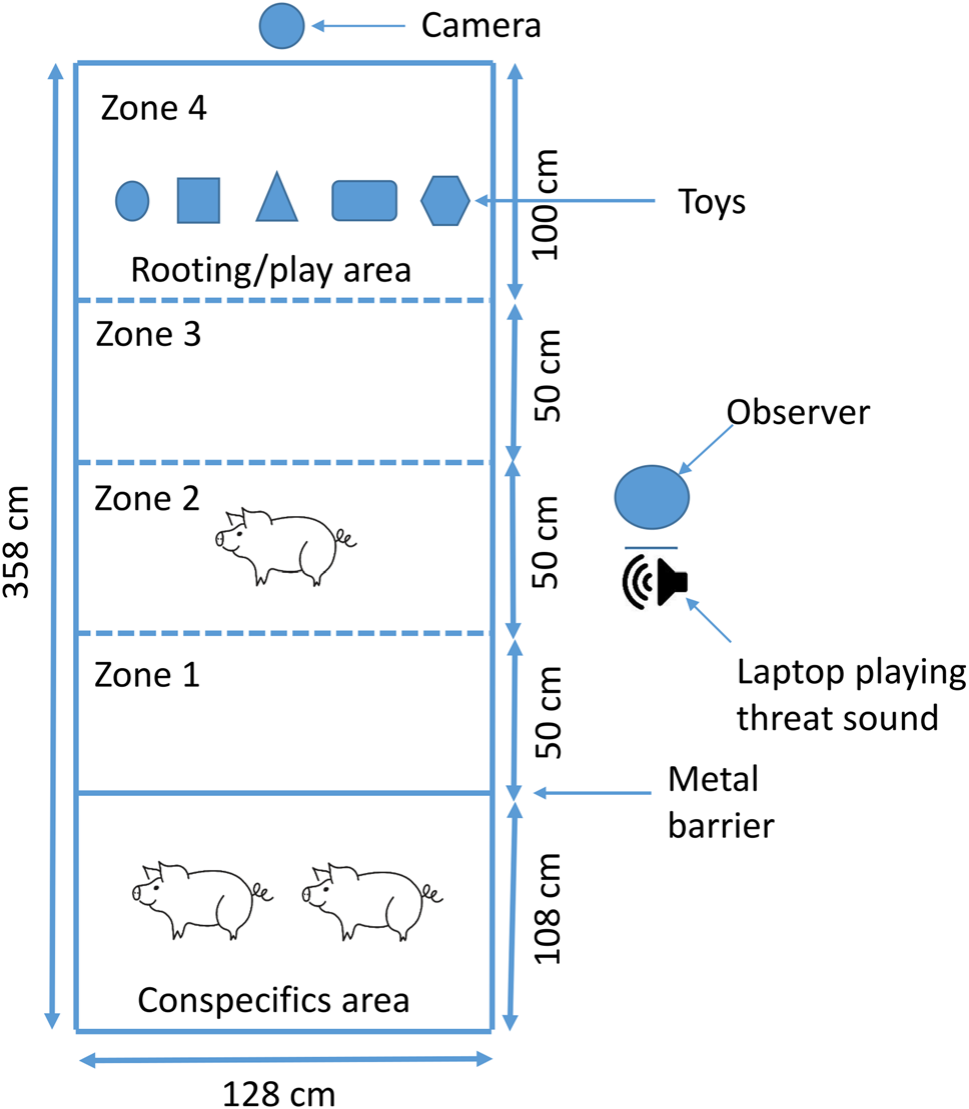
Attention bias arena. Pig vector from https://pixabay.com/vectors/pig-piglet-no-background-animal-2660356/.

The experimenters conducting the attention bias test were blind to the treatment of the piglets. An unfamiliar arena (Fig. 4) located in a different room (an empty pig stall) was used to assess attention bias. The arena consisted of three separate areas, a rooting and playing area bedded with straw and five different types of dog toys (placed from left to right: Ball, Hol-ee ball, Tire, Rope and Rope with rubber ring), a middle part (slatted metal floor) and a straw-bedded conspecifics area that was visible but not accessible to the test piglet. Two piglets (1 male and 1 female) from the same litter but not part of the experimental treatments were located in the conspecifics area in order to reduce isolation stress for the testing piglets. Thirty minutes before the start of the test, the conspecifics were habituated to the novel environment and stayed until all the piglets of the same litter had been tested. We observed that the conspecifics were either very calm or sleeping within 30 min of moving to the test arena, so it was assumed that 30 min was sufficient habituation. The gate separating the conspecifics area was made of metal bars that allowed visual, auditory and physical contact. The order in which the piglets within a litter were tested was random but balanced for treatment to ensure an even distribution of the treatments throughout the testing period (i.e., if the first tested piglet was a control, the second was a supplemented, the third was a control, etc.). In addition, four litters started with a randomly selected control piglet and three with a randomly selected supplemented piglet to control for any potential effects of being the first tested piglet. Random numbers were generated using the standard = RAND() function in Microsoft Excel.

The attention bias test was divided into two phases: a 90 s ‘before’ phase and a 90 s ‘after’ threat phase. The after phase started with a 15 s playback ‘threat’ sound of an aggressive dog bark. The piglets were raised indoors and had never seen a dog, and therefore this sound was most likely unfamiliar. Before the start of the test, the test piglet was gently lifted from its pen and placed into a trolley with straw and taken to the test arena. At time 0, it was placed in zone 2 (Fig. 4). It was then left to explore the arena for 90 s (before threat phase). At 90 s, the threat sound was played back from a computer located next to zone 2 and the piglet was left inside the arena for an additional 90 s (after threat phase). We did not control the piglet’s location in the arena when we played the threat sound, because if we had done that then we would not have been able to give the sound at the same exact time for each piglet.

The behaviours and locations described in the ethogram in Table 3, and the latency to the first time touching, or interacting with the toys were later analysed using the mangold interact software for behavioural analysis (Mangold International GmbH, Arnstorf, Germany) from video recordings by three different observer blind to the treatments. The agreement between observers was high (Kappa statistics between ranged between 0.77 and 1).

**Table 3:** Ethogram with behaviours scored during the attention bias test.

Behaviour (duration)	Definition
Standing	All four hooves are on the pen floor with limbs extended and without more than 1 step within 1 s
Walking	The piglet takes at least 2 steps within 1 s
Running	A fast paced movement involving all four legs
Vigilant	The head positioned at shoulder height or higher
Attention to threat	The head oriented towards the direction of the threat
Exploring	Sniffing or licking the floors, walls or pen fixtures
Interacting with toys	Touching, sniffing or manipulating toys
Conspecifics directed behaviours	Sniffing or licking the gate or positioning the snout between the bars
Rooting	Snout movement along the floor in the straw in area 3
Location	Time spent in zone 1, 2, 3 and 4

### Statistical analysis

Data were analysed using R (version 4.0.2) and R studio (version 1.4.1093)^71^. Not all periods were exactly 90 s for all animals (mean ± se were 95 ± 0.68 s for period 1 and 84 ± 0.70 s for period 2), and therefore a correction was applied to account for this (corrected variable = variable / actual period duration * 90 s). Normality and homoscedasticity assumptions were visually checked using QQ plots (LMERConvenienceFunctions package^72^). Variables that were not normally distributed were square root transformed and then it was checked if they met the assumptions of normality (variables: vigilance behaviour, exploring). However, the variable ‘exploring’ still did not meet normality assumptions, and therefore two observations with residuals greater than 2.5 were excluded from analysis, after which normality assumptions were met. In case data transformation was not sufficient and there were no clear outliers, a non-parametric Kruskal–Wallis test was used (variables: time spent in zones 1, 2, 3 and 4, interacting with toys, attention to threat) and the *p*-values were adjusted for multiple comparisons where necessary (time spent in the different zones). The duration of the variables described in Table 2 were initially analysed by linear mixed models (packages lme4^73^ and lmerTest^74^) with treatment, sex, phase and test order (and their interactions) as fixed effects and litter as a random effect. Non-significant terms and interactions were dropped in the final model. Post-hoc Tukey tests were performed with the package emmeans^75^. The data presented in Figs. 1, 2 and 3 was plotted using the package ggplot2 in R^76^.

## Data Availability

The datasets generated and analysed for the current study are available in the Open Science Framework repository: (https://osf.io/c9bfk/?view_only=328805b477ab4b2f95d956739fda87c9).
